# Tracking dyspnea up to supplemental oxygen prescription among patients with pulmonary fibrosis

**DOI:** 10.1186/s12890-017-0497-0

**Published:** 2017-11-22

**Authors:** Amy L. Olson, Bridget Graney, Susan Baird, Tara Churney, Kaitlin Fier, Marjorie Korn, Mark McCormick, David Sprunger, Thomas Vierzba, Frederick S. Wamboldt, Jeffrey J. Swigris

**Affiliations:** 1 Interstitial Lung Disease Program, National Jewish Health, Southside Building, Office #G011 1400 Jackson Street, Denver, CO 80206 USA; 2 Division of Pulmonary Sciences and Critical Care Medicine, University of Colorado Anschutz Medical Campus, Aurora, CO USA; 3 Participation Program for Pulmonary Fibrosis (P3F), Denver, CO USA; 4 Division of Pulmonary, Critical Care and Sleep Medicine, Sleep & Behavioral Health Sciences Section, National Jewish Health, Denver, CO USA

**Keywords:** Dyspnea, Hypoxia, Lung diseases, interstitial, Oxygen inhalation therapy, Quality of life

## Abstract

**Background:**

Dyspnea is the hallmark symptom of pulmonary fibrosis. Supplemental oxygen (O_2_) is prescribed to many patients with pulmonary fibrosis in hopes of alleviating dyspnea and improving physical functioning. We used response data from the University of California San Diego Shortness of Breath Questionnaire (UCSD) which was administered monthly in the context of a longitudinal, observational study to plot a rich trajectory for dyspnea over time in patients with pulmonary fibrosis. We used other data from that study to identify clinical predictors of being prescribed O_2_ and to provide additional information for how UCSD scores could be used for clinical purposes.

**Methods:**

We used linear mixed-effects models and multivariate Cox proportional hazards to model change in dyspnea scores over time and to identify significant predictors of time-to-O_2_-prescription among a pool of clinically-meaningful candidate variables. In the longitudinal study, all decisions, including whether or not to prescribe O_2_, were made by subjects’ treating physicians, not members of the research team.

**Results:**

One-hundred ninety-four subjects with pulmonary fibrosis completed more than one UCSD or were prescribed O_2_ at some point during the follow-up period (*N* = 43). Twenty-eight of the 43 had analyzable, longitudinal data and contribute data to the longitudinal UCSD analyses. All 43 were included in the time-to-O_2_-prescription analyses. Subjects prescribed O_2_ had more severe dyspnea at enrollment (38.4 ± 19.6 vs. 22.6 ± 18.7, *p* < 0.0001) and a steeper increase in UCSD scores over time (slope = 1.18 ± 0.53 vs. 0.24 ± 0.09 points per month, *p* = 0.02) than subjects not prescribed O_2_. Controlling for baseline UCSD score and FVC%, subjects with a clinical summary diagnosis of idiopathic pulmonary fibrosis (IPF) were far more likely to be prescribed O_2_ than subjects with other forms of pulmonary fibrosis (hazard ratio = 4.85, (2.19, 10.74), *p* < 0.0001).

**Conclusions:**

Baseline dyspnea and rise in dyspnea over time predict timing of O_2_ prescription. Accounting for disease severity, patients with IPF are more likely than patients with other forms of pulmonary fibrosis to be prescribed O_2_. UCSD scores provide clinically useful information; frequent administration could yield timely data on changes in disease status in patients with pulmonary fibrosis.

**Trial registration:**

The longitudinal study is registered on ClinicalTrials.gov (NCT01961362). Registered October 9, 2013.

**Electronic supplementary material:**

The online version of this article (10.1186/s12890-017-0497-0) contains supplementary material, which is available to authorized users.

## Background

Pulmonary fibrosis (PF) refers to a morphological pattern of interstitial lung disease in which the lung parenchyma is diffusely and irreversibly scarred [1]. There are many causes, but regardless of etiology, PF results in varying degrees of physiological restriction and impairment in gas exchange. The hallmark symptoms dyspnea, cough and fatigue, limit physical and social activities and substantially impair quality of life (QOL) [2, 3]. For most patients with PF, dyspnea is the strongest driver of QOL impairment: it forces them to slow down and avoid or give up activities they enjoy [4, 5].

As PF progresses, many patients develop hypoxemia. In parallel, symptoms—particularly exertional dyspnea and fatigue—increase and physical activity declines. Patients with PF qualify to receive supplemental oxygen therapy (O_2_) if blood oxygen level or peripheral oxygen saturation (SpO_2_) is found to be low while asleep or awake, either at rest or when physically active. The goals of O_2_ are to improve oxygenation (and avert complications of continuous or intermittent hypoxemia), decrease symptoms, increase mobility – and by extension, improve QOL.

Universally accepted consensus guidelines for idiopathic pulmonary fibrosis (IPF) suggest oxygenation and symptoms should be assessed at diagnosis and every follow-up evaluation [6]. No one would argue this is good practice for all patients with PF, regardless of etiology. Several indexes and questionnaires exist to quantify dyspnea, but it is unclear how useful they are in the clinical arena. In prior studies, we confirmed the University of California San Diego Shortness of Breath Questionnaire (UCSD) possessed validity to assess dyspnea in research studies of patients with IPF and showed how UCSD scores could be interpreted in a clinical context [7, 8].

For this analysis, we examined data from an observational cohort of patients with PF enrolled in a longitudinal, pre/post study designed to observe the effects of O_2_ on a range of patient-centered outcomes [9]. In the longitudinal study, no patients were on O_2_ at enrollment, and all treatment decisions, including whether to prescribe O_2_, were made by patients’ treating physicians, not the research team. Over the course of the longitudinal study, some patients were prescribed O_2_; many were not. Regardless, per the longitudinal study protocol, the UCSD was administered to subjects every month, starting at the time of enrollment and continuing to the end of the study. We aimed to achieve three objectives with this analysis: 1) to examine trends in monthly UCSD scores over time up until O_2_ was prescribed; 2) to identify significant predictors of O_2_ prescription from a pool of candidate clinical variables and 3) to improve understanding of the clinical implications of UCSD scores. We hypothesized monthly dyspnea assessments would predict the need for O_2_.

## Methods

### Subjects

The methods for the longitudinal study were published previously [9]. Briefly, between August 2013 and October 2015, patients with PF of any etiology, greater than 18 years of age, not using O_2_ and able to speak and read English, were recruited either from the Interstitial Lung Disease Clinic at National Jewish Health (NJH) or through our study website which is no longer active (www.pulmonaryfibrosisresearch.org). Diagnoses were confirmed by review of medical records and high-resolution chest computed tomography scans. For the many subjects followed outside of NJH, scans were mailed to our lab for review. Each subject gave written, informed consent. The study was approved by the NJH Institutional Review Board (HS-2790), and the study is registered on ClinicalTrials.gov (NCT01961362).

### Data capture and outcome measures

For the longitudinal study, data were collected at four time points: 1) enrollment; 2) 7-10 days prior to initiation of O_2_ (either continuously or with exertion); 3) 1 month after initiation of O_2_; and 4) 9–12 months after initiation of O_2_. In addition, the UCSD was obtained monthly after enrollment. For the primary analyses presented here, data included baseline characteristics and the monthly UCSD questionnaires up to time point 2 in subjects prescribed O_2_ or 15 months (in subjects who were not) among all subjects who completed at least two monthly UCSDs. We used REDCap (http://projectredcap.org/) to send and receive via email the UCSD each month.

The UCSD is a 24-item questionnaire with 21 items asking respondents to rate dyspnea while performing physical activities across a range of energy demands and 3 additional items that assess the impact of dyspnea [10]. UCSD scores range from 0 to 120; higher scores indicate greater dyspnea.

### Statistical analyses

Summary statistics were generated for baseline characteristics. To analyze UCSD scores over time, we developed linear mixed effects models using time, baseline UCSD and prescription of O_2_ (or not) as predictors. We included a random intercept for subjects. We assessed the most reasonable covariance structures for the repeated measures and found that unstructured (SAS Proc Mixed type = un) gave the best fit. The model yielded estimates for the parameters and for difference in slope for UCSD score between subjects who were ultimately prescribed O_2_ and those who were not prescribed O_2_ over the first 15 months after enrollment. We displayed plots for observed and predicted UCSD scores over time for the cohort stratified on whether O_2_ was prescribed. For exploratory purposes, we generated linear, spline-interpolated spaghetti plots of UCSD scores for the five subjects who died before being prescribed O_2_. Next, we examined predictors of time-to-O_2_ prescription over the course of the study by using multivariate Cox proportional hazards regression. Here, we included all 43 subjects prescribed O_2_, regardless of whether they had longitudinal UCSD data (i.e., more than one) or not. We first examined gender, age, smoking status, residence in Colorado (or not), IPF diagnosis (versus other), percent predicted forced vital capacity (FVC%), and baseline UCSD score in univariate analyses. Then, we used backward selection to determine a final model. Variables with *p* ≤ 0.2 in univariate analyses were considered for inclusion, and variables with *p* ≤ 0.05 were retained in the model. O_2_ prescription was the event. Subjects were followed to O_2_ prescription or, if they were not prescribed O_2_, then to last UCSD or death when, for this analysis, they were censored. In an exploratory analysis, we examined whether change in UCSD score would predict death regardless of whether O_2_ was prescribed. Here, time-to-death was the outcome and UCSD score was included in the model as a time-varying covariate and O_2_ prescription was included as a binary (yes/no) predictor. All analyses were performed using SAS version 9.3 statistical software (SAS, Inc.; Cary, NC).

## Results

One-hundred ninety-four subjects completed more than one UCSD or were prescribed O_2_. Forty-three were prescribed O_2_ at some point during the longitudinal study, and 166 were not. Of the 43 subjects prescribed O_2_, 28 had analyzable, longitudinal UCSD data. There was no difference in age, gender distribution, proportion with IPF, smoking history or FVC% between subjects with (*N* = 28) and subjects without (*N* = 15) longitudinal UCSD data (data not shown). Table 1 shows the baseline characteristics for the cohort stratified on whether O_2_ was prescribed. Compared to subjects not prescribed O_2_, those who were prescribed O_2_ had significantly greater physiological restriction (*p* = 0.008) and dyspnea (*p* < 0.0001) at enrollment.

**Table 1:** Baseline characteristics

	Prescribed O2 *N* = 28	Not Prescribed O2 *N* = 166	*P* value
Female	10 (36)	83 (50)	0.15
Age, yrs	67.5 ± 7.9	67.9 ± 9.6	0.99
Smoking			
Current	1 (4)	2 (1)	
Former	15 (54)	86 (52)	
Never	12 (43)	78 (47)	0.61
State of residence			
Colorado	4 (14)	67 (41)	
New Mexico	1 (4)	12 (7)	
Texas	3 (11)	12 (7)	
Other	20 (71)	75 (45)	0.03
Pulmonary fibrosis			
IPF	20 (71)	41 (25)	
CTD	4 (14)	62 (37)	
cHP	2 (7)	14 (8)	
FPF	1 (4)	11 (7)	
Other	1 (11)	38 (23)	< 0.0001
Surgical biopsy	11 (39)	59 (36)	0.61
FVC%	67.3 ± 17.5	77.81 ± 30.3^a^	0.008
DLCO%	52.5 ± 12.9	64.3 ± 15.4^b^	0.0016
Enrollment UCSD score	38.4 ± 19.6	22.6 ± 18.7	< 0.0001
Duration of follow-up, days	270.1 ± 201.5	473.6 ± 191.5	< 0.0001

^a^ *N* = 156 with FVC within 4 months of enrollment

^b^ *N* = 142 with DLCO within 4 months of enrollment

*IPF* idiopathic pulmonary fibrosis, *CTD* connective tissue disease-related pulmonary fibrosis, *cHP* chronic hypersensitivity pneumonitis, *FPF* familial pulmonary fibrosis; “Other” includes drug-induced, asbestosis, unclassifiable

**Fig. 1:**
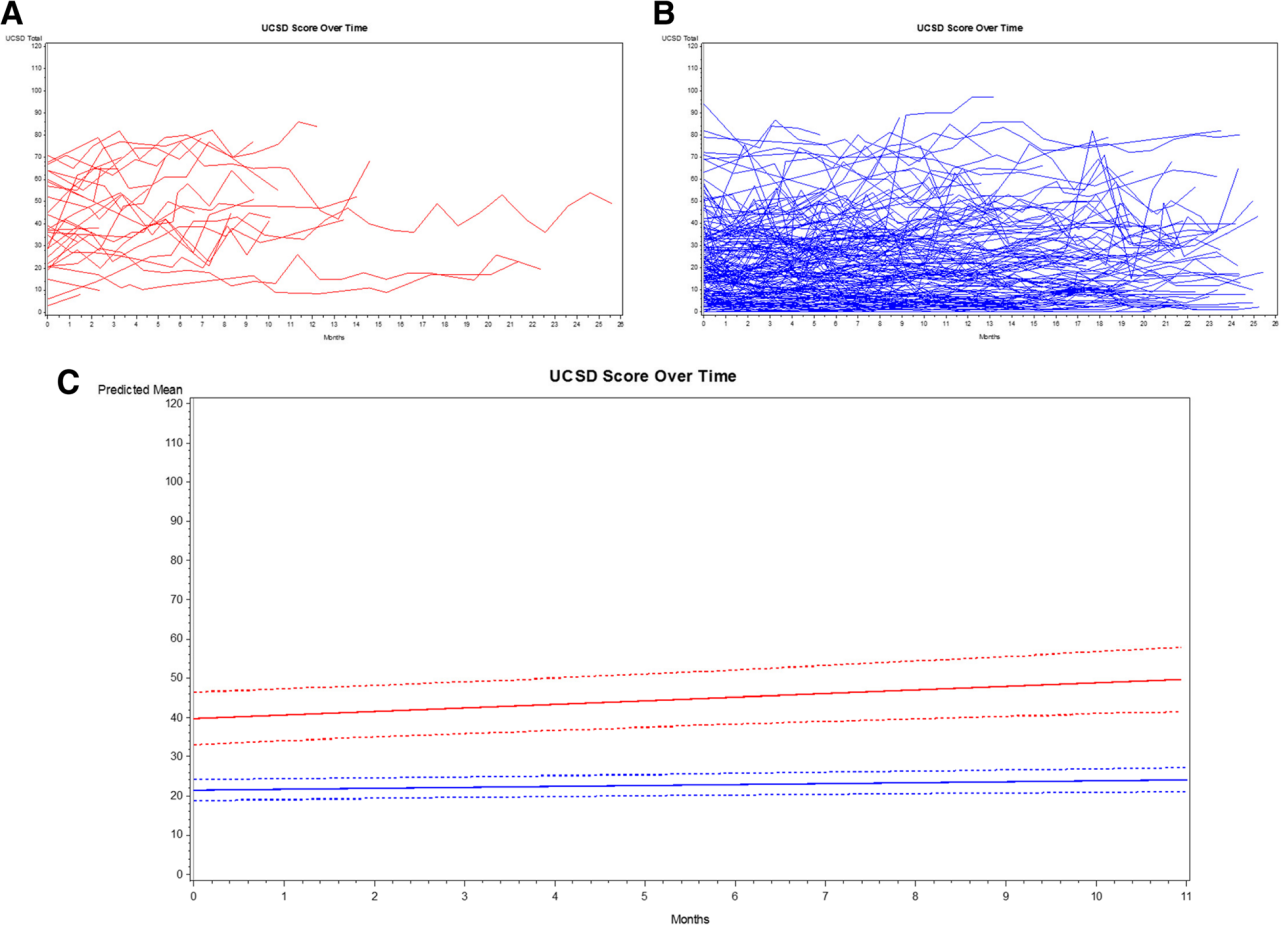
Panels **a**-**c** UCSD scores over time for subjects prescribed O2 (Panel **a**) or not (Panel **b**). Estimates for UCSD scores from mixed-effects model (Panel **c**). Footnote: In Panel **c**, estimate for subjects prescribed O2 red solid line, and estimate for subjects not prescribed O2 blue solid line. Dashed lines represent 95% confidence bands

In the mixed-effects model that included variables for baseline UCSD score and whether O_2_ was prescribed (Table 2), UCSD scores from subjects prescribed O_2_ rose more steeply than subjects not prescribed O_2_ (slope = 1.18 ± 0.53 vs. 0.24 ± 0.09 points per month, *p* = 0.02). Figure 1 shows spaghetti plots of UCSD scores for subjects prescribed O_2_ (Panel A) or not (Panel B) and a plot of model-generated estimates and confidence intervals for UCSD scores over time (Panel C). We built the same model but limited subjects to the subgroup with a clinical summary diagnosis of idiopathic pulmonary fibrosis (IPF), and the results were similar; among IPF patients prescribed O_2_, the slope was 1.97 ± 0.74 points per month vs. 0.22 ± 0.12 points per month among IPF patients not prescribed O_2_.

**Table 2:** Estimates for linear mixed effects model

	Estimate ± standard error	*p*
Intercept	1.18 ± 0.68	0.08
Time	0.24 ± 0.09	0.009
Prescribed O_2_	4.02 ± 1.32	0.002
Baseline UCSD	0.90 ± 0.02	< 0.0001

O_2_ = supplemental oxygen; UCSD = University of California San Diego Shortness of Breath Questionnaire

Univariate analysis of candidate predictors for time-to-O_2_-prescription are given in Table 3. Subjects who resided in Colorado were less likely to be prescribed O_2_ than subjects who resided in other states. Significant predictors of O_2_ prescription included IPF as the clinical summary diagnosis, lower FVC%, lower DLCO% and higher UCSD score at enrollment. The final multivariate model, after backward variable selection, included two predictors: 1) Baseline UCSD score, hazard ratio [HR] = 1.04, (1.02, 1.05), *p* < 0.0001; and 2) IPF clinical summary diagnosis, HR = 4.41, (2.26, 8.61), *p* < 0.0001. For interest, we forced baseline UCSD, IPF clinical summary diagnosis and baseline FVC% into a model, used no selection method and observed the following results: 1) Baseline UCSD score, HR = 1.04, (1.02, 1.06), p < 0.0001; 2) IPF clinical summary diagnosis, HR = 4.85, (2.19, 10.74), p < 0.0001; and 3) baseline FVC%, HR = 0.99 (0.97,1.01), *p* = 0.27).

**Table 3:** Predictors of being prescribed O_2_^a^

	Hazard Ratio (95% confidence limits)	*p*
Female	0.53 (0.28, 1.00)	0.05
Age, yrs	1.00 (0.97, 1.03)	0.99
Ever-smoker	1.62 (0.87, 3.04)	0.13
Reside in Colorado	0.24 (0.10, 0.61)	0.003
IPF	3.57 (1.93, 6.61)	< 0.0001
FVC%^b^	0.95 (0.93, 0.98)	0.0003
DLCO%^b^	0.96 (0.93, 0.99)	0.003
UCSD at enrollment	1.03 (1.01, 1.04)	< 0.0001

^a^For all 43 subjects prescribed O2, regardless of whether they completed UCSD prior to O2 prescription

^b^within 4 months of enrollment; IPF = idiopathic pulmonary fibrosis; FVC% = percent predicted forced vital capacity; UCSD = University of California San Diego Shortness of Breath Questionnaire

Five subjects died prior to being prescribed O_2_. Their observed median UCSD score at enrollment was 35.1 (22.0, 46.9). Their observed UCSD scores rose a median 36.0 (−9.9, 41.0) points over a median 295 (268, 319) days from enrollment to last UCSD collection. In a mixed-effects model that included variables for baseline UCSD score, subjects who died had slopes for UCSD scores that rose (3.8 ± 0.5 points/month) significantly more sharply than either subjects who were prescribed O_2_ (*p* < 0.0001) or subjects who were not prescribed O_2_ and survived (p < 0.0001). Spaghetti plots of UCSD scores over time for the five subjects who died are in the Additional file 1: Figure S1). In the exploratory survival analysis that included 10 subjects who died (5 prior to and 5 after O_2_ was prescribed), while adjusting for whether O_2_ was prescribed, any five-point increase in UCSD score was associated with a greater than 40% increase in the risk of death (HR = 1.44; 95% confidence limits 1.21, 1.73; *p* < 0.0001).

## Discussion

In this study, we analyzed dyspnea over time in a cohort of patients with PF enrolled in a longitudinal study. Compared with subjects who were not prescribed O_2_, those who were had more severe dyspnea at enrollment and a steeper increase in dyspnea scores over time.

To our knowledge, this is the first study in which such a rich trajectory of dyspnea has been plotted in patients with PF. In most studies and clinical trials, if dyspnea is assessed, it is done at 3-6 month intervals. Martinez and colleagues reported on UCSD scores collected every 12 weeks from 168 subjects in the placebo group of a randomized, controlled trial of interferon-ɣ1b for IPF [11]. They observed that mean UCSD scores rose minimally, from 45.1 at baseline to 46.8 at 72 weeks. However, among 36 subjects in the sample who died—although there was substantial variability—UCSD scores often rose sharply in the 12 weeks before death. The authors did not quantify these results, but a figure in their manuscript showing spaghetti plots for UCSD scores nicely displays the trends.

In our study, dyspnea scores from 4 of the 5 subjects who died before being prescribed O_2_ rose sharply in the 4-8 weeks prior to death. Among the 10 total subjects who died in the longitudinal study (5 before and 5 after being prescribed O2), an increase in dyspnea was associated with a 40% increase in the risk of death any time during the follow-up period. Unfortunately, we do not have data on their causes of death, how practitioners dealt with patients’ worsening symptoms—or whether they were even aware. Collecting quantitative dyspnea data more frequently from PF patients could potentially, like daily spirometry [12], alert patients and practitioners to PF acceleration, prompt urgent evaluation and trigger timely intervention. Future studies should examine whether dyspnea scores—and changes in them—alter therapeutic approaches and determine if they can be used to more accurately predict survival and other outcomes in patients with PF.

The results from this study reveal dyspnea predicts the outcome “being prescribed O_2_”, something perceived by PF patients and their caregivers as a major setback—as a potentially activity-limiting and socially constraining intervention that impacts quality of life [3, 4]. We can extrapolate our results to add clinical context: using the parameter estimate for UCSD at enrollment from the univariate Cox proportional hazards model (0.026), any 8-point increase in enrollment UCSD score (a point estimate for it’s minimum important difference [7]) is associated with a 23% increased risk of being prescribed O_2_ in the ensuing 15 months. The protective effect of living in Colorado is due to them being enrolled in the study earlier than other subjects in the course of illness.

In previously published work from our lab, we created a 21-item UCSD Dyspnea Ruler that can also put UCSD scores in a clinical context [8]. To use the Ruler, one sums the responses from the first 21 items on the UCSD. These items ask respondents to rate their dyspnea severity while performing physical activities that span a range of energy demands (determined by the activity’s metabolic equivalents or METS). As PF progresses, respondents become more likely to rate dyspnea as worse for a given activity. For example, a patient with mild PF might rate their dyspnea as mild when walking up a flight of stairs, but if PF progresses, their dyspnea rating for this roughly 8-MET activity would increase. We calculated 21-item UCSD scores for subjects in the current study: the Dyspnea Ruler would predict that, at baseline, subjects not prescribed O_2_ during the course of the study would have described moderate dyspnea for the UCSD item “How short of breath do you get walking up a hill?” (i.e., they would have an equal probability of responding “2” or “3” on the 0-5 scale). Meanwhile, at baseline, subjects prescribed O_2_ during the course of the study would describe the same level of moderate dyspnea while doing a less strenuous task, such as washing a car (or carrying a light [e.g., 15-pound] load on level ground) [13]. By the time they were prescribed O_2_, they would have described moderate dyspnea with even less strenuous activities, like walking downstairs, clearing the table after a meal, or walking leisurely to a neighbor’s house for social reasons [13].

We observed the two strongest, independent predictors of being prescribed O_2_ were dyspnea severity at enrollment and an IPF clinical summary diagnosis. In a model controlling for enrollment UCSD and FVC%, subjects with IPF were over four times more likely than subjects with other diagnoses to be prescribed O_2_. The fact that dyspnea increased significantly in many (but not all) subjects with IPF is not surprising. That subjects with IPF were more likely than other subjects to have been prescribed O_2_ is reassuring. To us, it signals that practitioners are recognizing the likelihood of disease progression and hypoxemia in patients with IPF; and they are assessing/reassessing oxygen status and responding to abnormal results. Whether O_2_ prescription translates to improvements in the way patients with IPF (or PF of any cause) feel or function, or in how long they survive, are presently questions without answers and topics for other studies.

This observational study has limitations. In an attempt to offer enrollment to as many patients as possible, we designed a nationwide study in which patients were not required to travel, even to their local physician or medical facility. This made data collection and diagnosis confirmation challenging but not impossible. We did our best to review all available medical records, including chest computed tomography images, to assess the fidelity of diagnoses recorded in subjects’ medical records. We did not request surgical biopsy slides for review. Having incomplete historical data could have introduced misclassification bias; however, because subjects were enrolled prior to being prescribed O_2_, if present, misclassification would have been nondifferential—equally present in both subgroups (prescribed O_2_ and not prescribed O_2_). Nondifferential misclassification would bias toward the null, diluting between-groups differences.

Of 300 subjects enrolled, only 194 had more than one monthly UCSD data collection, and of these, O_2_ was prescribed in 28. Despite the low number of subjects receiving O_2_, administering the UCSD monthly yielded a large number of data points, giving precision to many model estimates—like change in UCSD over the first 12 months.

The research team played no role in determining whether or when subjects were prescribed O_2_. This lack of autonomy over O_2_ prescription precludes us from drawing firm conclusions on precisely when and whether patients needed O_2_, how the determination of need for O_2_ was made, or when or how it was prescribed if needed. We were merely collecting data before and after. The real value of this analysis lies in its “real-world”, patient-centered design and the rich dataset resulting from monthly dyspnea data collection.

Many other factors, both measurable and unmeasurable, likely affected when and whether O_2_ was prescribed. Measurable, patient-related characteristics for which we have no data include insurance coverage, economic status, access to care, proximity to O_2_ suppliers, and patients’ values and preferences for their personal health and healthcare. Among others, unknown practitioner-related factors include their beliefs and judgments about the potential merits of O_2_ for patients with PF.

## Conclusion

Among patients with PF, dyspnea levels are associated with timing of O_2_ prescription. Baseline dyspnea levels can predict the need for O_2_ in the near future. Controlling for the severity of physiological restriction and dyspnea level, patients with IPF are more likely to be prescribed O_2_ than patients with PF from other causes. Research is needed to better understand this phenomenon, to determine the effects of O_2_ on how patients with PF feel and function, and to ascertain whether frequent dyspnea assessments could be used to alter therapeutic interventions and outcomes in PF.

## Additional file

Additional file 1: Spaghetti plots of UCSD scores for five subjects who died before being prescribed supplemental oxygen. (DOCX 40 kb)
