# Supplementary material for: Tracking dyspnea up to supplemental oxygen prescription among patients with pulmonary fibrosis
Source: BMC Pulm Med. 2017 Nov 22;17:152. doi: 10.1186/s12890-017-0497-0 (PMC5700736; doi:10.1186/s12890-017-0497-0)
Supplement: Additional file 1: — Spaghetti plots of UCSD scores for five subjects who died before being prescribed supplemental oxygen. (DOCX 40 kb) [file 12890_2017_497_MOESM1_ESM.docx]

**Additional file 1**

**Tracking dyspnea up to supplemental oxygen prescription**

**among patients with pulmonary fibrosis**

^1, 4^Amy L. Olson, MD; Bridget Graney, MD^2,3^, Susan Baird^3^, Tara Churney, MPH^1,3^, Kaitlin Fier, MPH^3^, Marjorie Korn^3^, Mark McCormick^3^, David Sprunger, MD^1,4^, Thomas Vierzba^3^, Frederick S. Wamboldt, MD^3,4^, Jeffrey J. Swigris, DO, MS^1,3,4^

^1^Interstitial Lung Disease Program, National Jewish Health, Southside Building, Office #G011 1400 Jackson Street Denver, CO 80206, USA; ^2^Division of Pulmonary Sciences and Critical Care Medicine, University of Colorado Anschutz Medical Campus, Aurora, CO USA; ^3^Participation Program for Pulmonary Fibrosis (P3F), Denver, CO, USA; ^4^Division of Pulmonary, Critical Care and Sleep Medicine, Sleep & Behavioral Health Sciences Section, National Jewish Health, Denver, CO, USA

**Figure S1. Sp**aghetti plots of UCSD scores for five subjects who died before being prescribed supplemental oxygen.

**Footnote:** Lines show UCSD scores over time for each of five subjects. As in the paper by Martinez et al. (reference #11), a smoothing function was applied.
